# Evidence produced in Japan: tegafur-based preparations for postoperative chemotherapy in breast cancer

**DOI:** 10.1007/s12282-013-0451-9

**Published:** 2013-03-01

**Authors:** Toru Watanabe

**Affiliations:** Department of Medicine, Hamamatsu Oncology Center, 3-6-13 Chuo, Naka-ku, Hamamatsu, Shizuoka 430-0929 Japan

**Keywords:** Breast cancer, Adjuvant, Fluorouracil, Tegafur, Review

## Abstract

Oral fluoropyrimidine anticancer agents (oral 5-fluorouracil [5-FU]) able to be used as chemotherapy for breast cancer include tegafur–uracil (UFT), tegafur–gimeracil–oteracil potassium (S-1), doxifluridine, and capecitabine. Since the 1980s, UFT has been most widely used for postoperative chemotherapy in breast cancer. UFT is an oral preparation that was designed to achieve and maintain high concentrations of 5-FU in plasma by combining tegafur, a prodrug of 5-FU, with uracil. UFT is characterized by mild adverse events, allowing long-term treatment. The prolonged maintenance of high plasma 5-FU concentrations has been suggested to inhibit micrometastases after surgery. Recently, large clinical trials conducted in Japan have shown that UFT-based postoperative chemotherapy is therapeutically useful in patients with node-negative (n0), high-risk breast cancer. We review the results of clinical trials of postoperative chemotherapy with UFT in Japan and discuss its roles and future prospects.

## Introduction

In their guidelines for treatment selection according to risk category, the St. Gallen International Conference recommend endocrine therapy alone or chemotherapy followed by endocrine therapy for postoperative adjuvant chemotherapy in patients with estrogen receptor (ER)-positive, human epidermal growth factor receptor 2 (HER2)-negative breast cancer [1]. The National Comprehensive Cancer Network (NCCN) guidelines also recommend similar treatment options. However, among patients with ER-positive, HER2-negative breast cancer, it remains to be clarified which chemotherapeutic regimens are most effective in what types of patients.

At present, combinations of drugs such as anthracyclines and taxanes are used as standard postoperative chemotherapy in patients with breast cancer. However, recent studies have suggested that these standard chemotherapeutic regimens provide limited therapeutic effectiveness in patients with ER-positive or HER2-negative breast cancer. Awareness that conventional standard anticancer agents are not necessarily adequate for the management of ER-positive, HER2-negative breast cancer has led to the search for new treatment strategies.

## Fluoropyrimidine derivatives used as chemotherapy for breast cancer

In 1956, 5-fluorouracil (5-FU), a fluoropyrimidine, was synthesized by Heidelberger et al. and Duschinsky et al. This drug continues to play a central role in chemotherapy of solid tumors and is an important component of combination chemotherapy regimens for breast cancer. In breast cancer, 5-FU is mainly given as a bolus injection in regimens such as cyclophosphamide, methotrexate, and 5-FU (CMF) and 5-FU, epirubicin, and cyclophosphamide (FEC). Experimental studies have suggested that divided, low doses of 5-FU have a greater impact on survival than a large single bolus dose [2].

However, 5-FU has the disadvantage that 85 % of the administered dose is promptly catabolized and inactivated by dihydropyrimidine dehydrogenase (DPD), similar to uracil. Derivatives of 5-FU have been developed to overcome this disadvantage and to enhance antitumor efficacy.

**Fig. 1:**
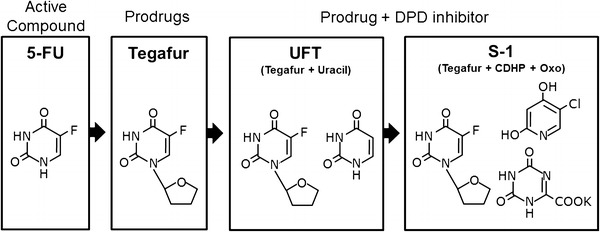
Development of oral formulations. *5-FU* 5-fluorouracil, *DPD* dihydropyrimidine dehydrogenase, *UFT* tegafur plus uracil, *TS-1* tegafur, CDHP (gimeracil), and Oxo (oteracil)

Tegafur, a prodrug of 5-FU, was synthesized in 1967 for injectable use. Subsequently, tegafur was found to be well absorbed after oral administration, resulting in prolonged plasma concentrations of tegafur and 5-FU. These findings led to the development of oral formulations (Fig. 1).

Oral 5-FU preparations developed in Japan were approved on the basis of the results of clinical trials in women with metastatic or recurrent breast cancer. After entering the 1990s, oral formulations of 5-FU have been widely used as postoperative chemotherapy for breast cancer because of convenience of administration and low incidences of serious adverse events. At that time, however, the Adjuvant Chemoendocrine Therapy for Breast Cancer (ACETBC) trial was in progress to confirm the effectiveness of UFT as postoperative chemotherapy for breast cancer, and this drug had been used in general clinical practice without adequate clinical evidence of efficacy. Moreover, CMF, an internationally accepted standard therapy, was not used in Japan at that time. Therefore, the National Surgical Adjuvant Study of Breast Cancer 01 (N·SAS-BC01) trial was started as a clinical research project supported by the Japanese Ministry of Health, Labor, and Welfare to compare UFT with CMF. Around the same time as this trial, the Study Group for the Comparative Trial with UFT + Tamoxifen and CMF + Tamoxifen in Adjuvant-therapy for Breast Cancer (CUBC) trial [3–7] was also started to compare UFT with CMF.

We review the results of clinical trials of UFT performed in Japan and discuss its roles and future prospects.

## Postoperative chemotherapy with UFT in breast cancer

UFT is a preparation combining tegafur, a prodrug of 5-FU, with uracil. However, after tegafur is metabolically converted into 5-FU, 5-FU is promptly catabolized by DPD in the liver, similarly to injected 5-FU. Therefore, uracil, a competitive inhibitor of DPD, was combined with tegafur to find ways to increase plasma 5-FU concentrations and enhance antitumor activity. The optimal combination ratio of tegafur to uracil was found to be 1:4 (molar ratio), taking into account the balance between efficacy and safety. This molar ratio was applied to UFT. In addition, metabolites of tegafur such as γ-hydroxybutylate (GHB) and γ-butyrolactone (GBL) have been reported to inhibit angiogenesis. Treatment with UFT is thus thought to have 5-FU-induced cytocidal effects on cancer cells remaining after surgery as well as inhibitory effects on angiogenesis, produced by GHB and GBL [8, 9].

In Japan, clinical trials have been conducted to evaluate postoperative chemotherapy with oral 5-FU preparations in patients with early breast cancer. In the third ACETBC trial, which studied the effect of UFT in patients with stage I–IIIa resected breast cancer, additional treatment with UFT was shown to improve the 5-year relapse-free survival rate [hazard ratio of the UFT group to the control group, 0.77; 95 % confidence interval (CI), 0.60–0.99]. In the fourth ACETBC trial, patients with axillary node-negative breast cancer were randomly assigned to 4 treatment groups: surgery alone, postoperative treatment with tamoxifen for 2 years, postoperative treatment with UFT for 2 years, and postoperative treatment with tamoxifen plus UFT for 2 years. An analysis of outcomes according to the presence or absence of treatment with UFT showed that UFT significantly improved survival rates. In particular, among patients with ER-positive breast cancer, the survival rate was highest in the tamoxifen plus UFT group (hazard ratio of the tamoxifen plus UFT group to the surgery alone group, 0.28; 95 % CI 0.085–0.93).

Histopathological specimens were retrieved from the surgical-pathology files for premenopausal women with ER-positive, axillary-node-positive breast cancer who were enrolled in the third ACETBC trial (tamoxifen vs. tamoxifen plus UFT). The tumor specimens were stained immunohistochemically to investigate the relation between HER2 expression status and the inhibitory effect of UFT on recurrence. The results suggested that the additive effect obtained by combining UFT with tamoxifen was unaffected by HER2 expression status [10]. UFT is therefore expected to inhibit recurrence even in patients with HER2-negative breast cancer.

## Evidence on oral fluoropyrimidine preparations as compared with CMF control therapy

### N·SAS-BC01 trial

CMF therapy in Japan is far behind that in Western countries, and was approved in 1996. After approval, nationwide clinical trials with a CMF control group were performed in Japan. One of these studies examined the noninferiority of UFT to CMF.

**Fig. 2:**
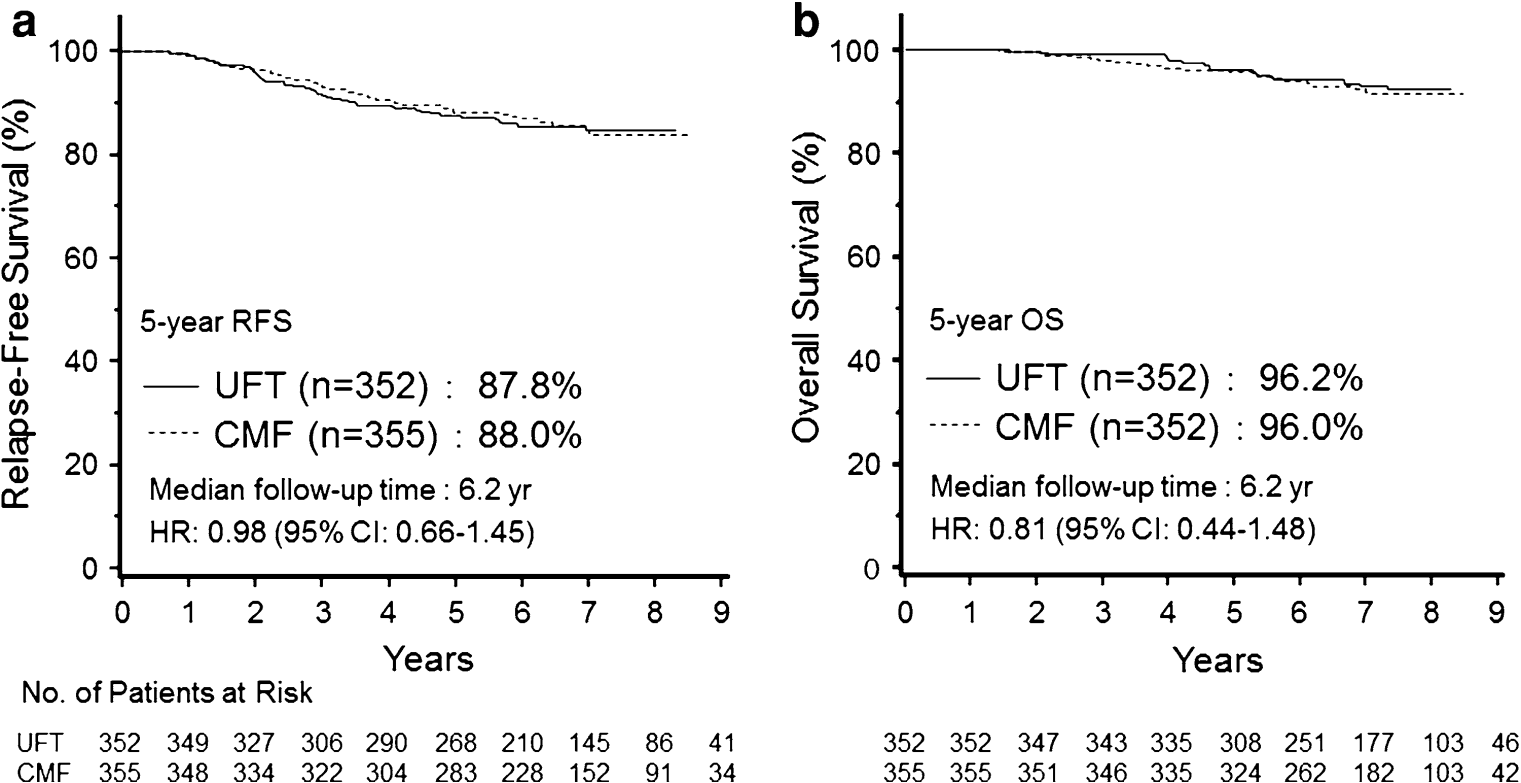
Relapse-free survival (RFS) and overall survival (OS) in the N·SAS-BC01 trial. **a** RFS and **b** OS of the total patients treated with uracil and tegafur (UFT) or cyclophosphamide, methotrexate, and fluorouracil. *CMF* cyclophosphamide, methotrexate, and 5-fluorouracil, *HR* hazard ratio, *CI* confidence interval

The N·SAS-BC01 trial was designed to establish the noninferiority of UFT to CMF in patients with node-negative (n0), high-risk breast cancer. The primary endpoint was relapse-free survival. Six cycles of classic CMF, given to the control group, were compared with 2 years of treatment with UFT. Patients whose tumors were positive for ER, progesterone receptor, or both concurrently received tamoxifen (20 mg/day) for 5 years. A total of 733 patients were enrolled in both groups combined. The hazard ratio of the UFT group to the CMF group was 0.98 (95 % CI 0.66–1.45). In patients with n0 high-risk breast cancer, the noninferiority of the UFT group to the CMF group was not demonstrated statistically, but the relapse-free survival curves and survival curves appear to be superimposable, strongly suggesting that both treatments are similarly effective [5] (Fig. 2). Grade 3 or 4 adverse events occurred in fewer than 10 % of the patients in both arms. The incidence of grade 3 or 4 leucopenia was significantly higher in the CMF arm, and the incidences of grade 3 or 4 diarrhea, anemia, elevated AST, and elevated serum total bilirubin were significantly higher in the UFT arm. The incidence of alopecia (any grade) was lower in the UFT group (9.7 %) than in the CMF group (55.2 %).

### CUBC trial

The CUBC trial was designed to demonstrate the noninferiority of UFT to CMF in patients with stage I–IIIa breast cancer who had 1–9 metastatic axillary nodes. It was performed at the same time as the N·SAS-BC01 trial. The CUBC trial compared 6 cycles of CMF plus 2 years of tamoxifen with UFT plus tamoxifen, given concurrently for 2 years. On analysis of data on 377 enrolled patients, the 5-year relapse-free survival rate was similar in the CMF group (76.3 %) and the UFT group (72.3 %). Adverse events were reported in 88.1 % (156 out of 177) of patients receiving UFT and in 98.8 % (171 out of 173) of those receiving CMF, showing a significantly lower incidence in the UFT group (*P* = 0.05). The incidence of leukopenia as well as hemoglobin, anorexia, nausea/vomiting, stomatitis, and alopecia was significantly higher in the CMF group, whereas that of liver dysfunction was significantly higher in the UFT group. A subset analysis according to hormone-receptor status showed that the 5-year relapse-free survival rate was better in the CMF group than in the UFT group among patients with ER-negative tumors. Among patients with ER-positive tumors, however, the 5-year relapse-free survival rate was better in the UFT group (81 %) than in the CMF group (76 %) (hazard ratio of the UFT group to the CMF group, 0.73; 95 % CI 0.38–1.39). These findings indicated a trend toward an interaction between ER-receptor status and therapeutic effectiveness [7].

**Fig. 3:**
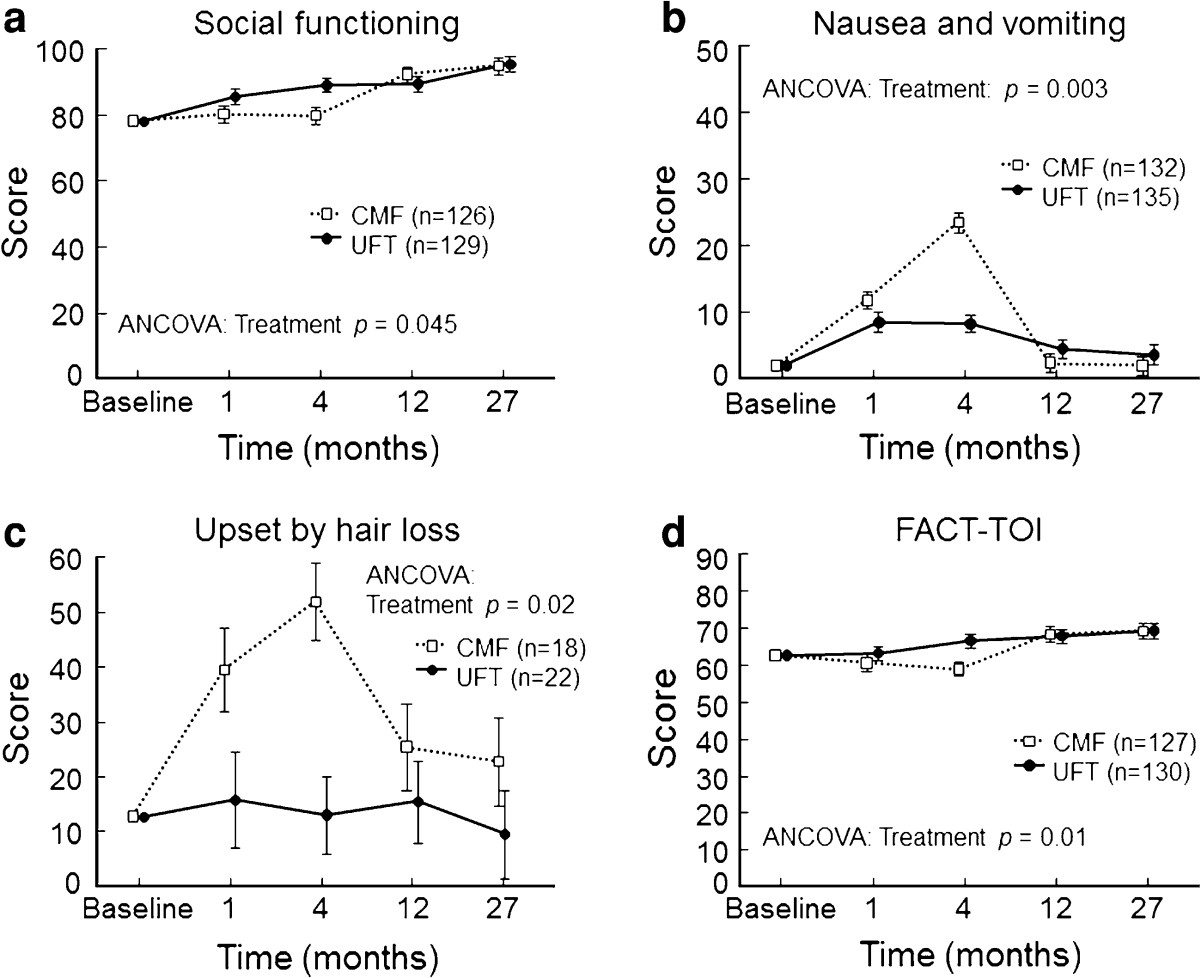
Impact of UFT or CMF on QOL in patients taking part in the N·SAS-BC 01 trial. European Organization for Research and Treatment of Cancer Quality of Life Questionnaire Core 30 Breast 23 scores for social functioning (**a**), nausea and vomiting (**b**), and upset by hair loss (**c**). In the graph for social functioning, a higher score indicates better QOL, whereas for nausea and vomiting and upset by hair loss, lower scores indicate better QOL. **d** FACT-TOI. A higher score indicates better QOL. Data are presented as mean standard error

Oral 5-FU is known to be associated with few adverse events such as gastrointestinal symptoms, myelosuppression, and hair loss. Clinical studies performed to date have shown a similar trend for UFT. The N·SAS-BC01 trial used the European Organization for Research and Treatment of Cancer (EORTC) QLQ C30, QLQ-BR23, and the Functional Assessment of Cancer Therapy-Breast (FACT-B) questionnaires to evaluate patients’ quality of life (QOL). The results of QOL analyses showed that QOL was distinctly better in the UFT group than in the CMF group for about 6 months after the start of treatment. After the completion of CMF therapy, QOL improved, but was similar to that in the UFT group. These findings indicated that a good QOL was maintained during treatment with UFT (Fig. 3).

## Meta-analysis of the N·SAS-BC01 and the CUBC trials

**Fig. 4:**
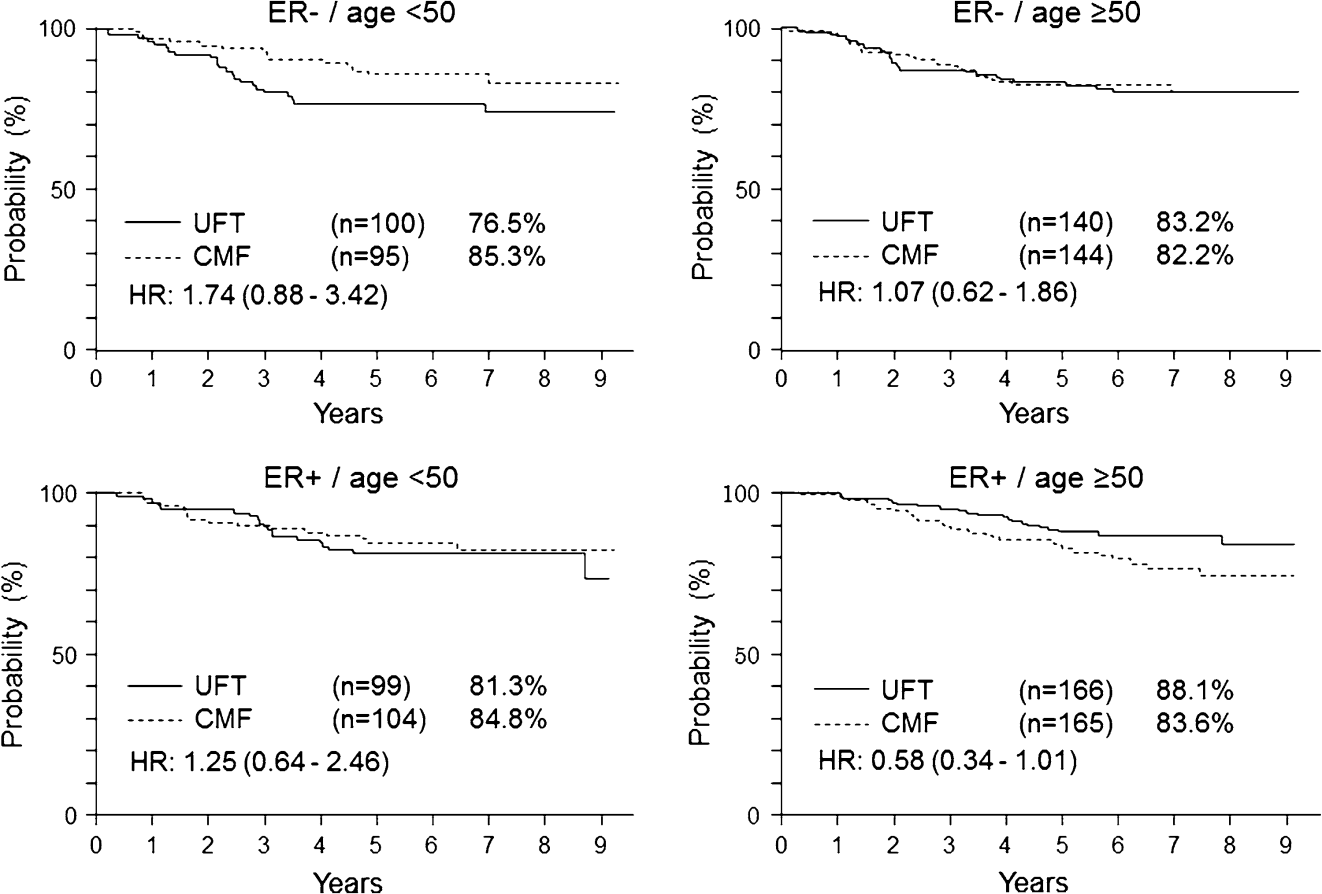
Relapse-free survival according to estrogen receptor (ER) and age in the pooled analysis of N·SAS-BC01 trial and CUBC trial. *HR* hazard ratio

The results of a pooled analysis of the N·SAS-BC01 trial and the CUBC trial, both of which compared UFT with CMF, have been reported [11]. The noninferiority of UFT to CMF was not demonstrated statistically in the study group as a whole. In patients with ER-positive tumors, however, the noninferiority of UFT to CMF was statistically proven (hazard ratio of the UFT group to the CMF group, 0.79; 97.5 % CI 0.49–1.27). A subgroup analysis showed UFT was particularly more effective than CMF in patients 50 years or older who had ER-positive tumors (hazard ratio of the UFT group to the CMF group, 0.58; 95 % CI 0.34–1.01). These results suggested that UFT combined with endocrine therapy can effectively inhibit recurrence in patients with ER-positive breast cancer (Fig. 4).

## Expectations for S-1

S-1 is a preparation that was developed to achieve higher antitumor activity than UFT with less toxicity. To reach this goal, potent DPD inhibitors and agents designed to reduce adverse events were studied. Gimeracil, a potent DPD inhibitor about 200-fold more active than uracil, was developed [12]. To reduce gastrointestinal toxicity, oteracil potassium was discovered. This drug inhibits activation of 5-FU in the gastrointestinal tract, reducing gastrointestinal toxicity. To achieve a good balance between efficacy and toxicity, optimal ratios for combining these 2 components with tegafur, a prodrug of 5-FU, were studied. Consequently, tegafur, gimeracil, and oteracil potassium were combined at a molar ratio of 1:0.4:1 in S-1.

As for clinical outcomes in breast cancer, the results of 3 clinical studies of S-1 in patients with advanced or recurrent breast cancer have been reported. Response rates were 40.7 and 42.0 % in patients who received S-1 as first- or second-line treatment, respectively [13], as compared with 21.8 % in patients who did not respond to anthracycline or taxanes. When UFT was given as first-line treatment, the response rate was 32 % in patients with advanced or recurrent breast cancer. The response rate with UFT plus leucovorin calcium has been reported to range from 10 to 13.2 % in patients who did not respond to anthracyclines or taxanes. S-1 can be expected to have higher antitumor activity than UFT [14–16].

At present, the Post Operative Therapy with Endocrine and TS-1 (POTENT) trial, a randomized, controlled, phase III study of postoperative chemotherapy in patients with ER-positive, HER2-negative breast cancer, is ongoing.

## Discussion

With recent progress in drug therapy, the primary treatment of early breast cancer has shifted from surgery to multidisciplinary treatment, including drug therapy. During the approximately 30 years since CMF was reported to be therapeutically useful for postoperative chemotherapy, many randomized, controlled clinical trials and meta-analyses of their results have dramatically changed the management of breast cancer, especially postoperative chemotherapy. In 1996, classic CMF, designated as standard therapy in Western countries, was approved in Japan. Randomized, controlled clinical trials comparing the oral 5-FU preparation UFT with classic CMF therapy were planned and conducted, leading to the introduction of standard therapy used in Western countries to Japan.

At present, anthracycline- and taxane-based combination regimens are mainly used as standard chemotherapy for the postoperative management of breast cancer. However, recent studies suggest that standard chemotherapy with anthracyclines and taxanes is less effective in patients with ER-positive or HER2-negative breast cancer. A meta-analysis of 8 studies (5,354 patients) evaluating the efficacy of postoperative adjuvant therapy with anthracycline derivatives according to HER2 expression status suggested that anthracyclines are only marginally beneficial in patients with HER2-negative tumors [17]. A subset analysis of the Cancer and Leukemia Group B (CALGB) 9344 trial, which evaluated the additive effect of paclitaxel after doxorubicin (Adriamycin) plus cyclophosphamide (AC) in postoperative patients with breast cancer, reported that additional treatment with paclitaxel was ineffective in patients with ER-positive, HER2-negative tumors [18]. The UK-based Taxotere as Adjuvant Chemotherapy Trial (UK-TACT) studied the effect of adding docetaxel to FEC. However, additional treatment with docetaxel was similarly found to be ineffective in patients with ER-positive, HER2-negative breast cancer [19].

ER-positive, HER2-negative breast cancer is thus less sensitive to chemotherapy. In addition, there are no clear-cut clinical guidelines for deciding whether a patient should receive endocrine therapy alone or combined with chemotherapy. Treatment selection is also complicated by the fact that a patient’s diagnosis may be upgraded from low risk to intermediate risk because of the presence of only a single intermediate risk factor. Concern that additional postoperative chemotherapy may negatively affect patients’ QOL by causing hair loss and other adverse events also influences treatment decisions. Another important factor is that intensive intravenous chemotherapy is unsuitable for some patients because of advanced age, concurrent disease, or other factors.

Recently, increasing emphasis has been placed on assigning treatment policies according to the biologic characteristics of tumors (molecular subtype), broadly classified into 4 subtypes: luminal A, luminal B, triple-negative, and HER2. Patients with triple-negative breast cancer currently receive cytotoxic chemotherapy because they lack distinct treatment targets. Drugs with various molecular targets are currently being developed and are expected to improve treatment outcomes. The treatment of HER2-positive breast cancer with trastuzumab, an anti-HER2 agent, has considerably improved outcomes. Besides trastuzumab, various types of drugs are scheduled to be launched and are eagerly awaited.

Luminal A or B breast cancer is hormone sensitive and treated by endocrine therapy. Chemotherapy is additionally given to patients with a high baseline risk of recurrence. Because luminal A breast cancer is considered to have relatively low proliferative activity of tumor cells and low sensitivity to conventional chemotherapy, endocrine therapy is the mainstay of treatment. However, some patients have a slightly increased baseline risk of recurrence. What types of chemotherapeutic regimens should be additionally given to such patients remains controversial. Luminal B breast cancer is often associated with relatively high proliferative activity and is frequently treated by chemotherapy in addition to endocrine therapy. These two subtypes of breast cancer are not only associated with early postoperative recurrence, but also with late recurrence 5 or more years after surgery.

The type of chemotherapy should be decided on the basis of baseline risks and patients’ preferences. In Japan, UFT has been shown to be noninferior to CMF in postmenopausal women with hormone-sensitive breast cancer. In contrast to conventional intravenous chemotherapy, UFT is well tolerated and has become an important treatment option supported by extensive evidence of efficacy.

UFT was developed in Japan. In particular, UFT differs from other cytotoxic anticancer agents because UFT can be used concurrently with endocrine therapy and can be administered for a prolonged period. Experimental studies have provided evidence that oral 5-FU is useful in combination with tamoxifen. Concurrent use of 4-OH-tamoxifen and 5-FU has been shown to have additive antitumor activity [20]. As for the mechanism involved, tamoxifen has been reported to lower the activity of TS, a key enzyme in the inhibition of DNA synthesis by 5-FU, thereby enhancing the antitumor activity of 5-FU. In studies assessing the combined effectiveness of UFT and an aromatase inhibitor in cell lines with induced aromatase expression, concurrent treatment with both drugs was confirmed to significantly decrease tumor volume compared to either drug alone [21].

Recent studies of chemotherapy combined with endocrine therapy have reported that aromatase-inhibitor-based endocrine therapy plus chemotherapy is useful [22, 23]. In addition, GHB and GBL derived from tetrahydrofuran metabolites specific to tegafur have been shown to inhibit angiogenesis. Therefore, long-term, metronomic treatment with UFT may produce high antitumor activity [24]. Long-term treatment with UFT may continuously inhibit tumor angiogenesis, suppressing postoperative metastasis.

Although evidence derived from clinical studies supporting the combined use of UFT and an aromatase inhibitor is currently unavailable, these drugs are often combined in clinical practice. In a survey primarily designed to confirm the tolerability of 1-year postoperative treatment with UFT [25], UFT was given from 2002 through 2005. Among 1,995 patients in whom safety was assessable at 1 year, 273 concurrently received UFT plus anastrozole, 398 received UFT alone, and 127 received UFT plus tamoxifen. Treatment in these 3 groups was confirmed to be safe and adequately tolerable. As mentioned above, experimental studies have shown that concurrent treatment with UFT and anastrozole results in higher antitumor activity than either UFT or anastrozole alone. On the basis of available evidence, combined use of UFT plus an aromatase inhibitor is thus considered a viable option for postoperative adjuvant chemotherapy in addition to the evidence-based combination of UFT plus tamoxifen.

Other oral 5-FU derivatives studied as postoperative chemotherapy include capecitabine and doxifluridine, a metabolite of capecitabine. Like UFT, doxifluridine has been mainly evaluated as postoperative chemotherapy in Japan; however, its usefulness as compared with surgery alone could not be demonstrated [26]. The CALGB/CTSU 49907 trial compared capecitabine, a more tumor-selective drug than doxifluridine, with standard control treatment comprising CMF or AC in older patients with breast cancer. Capecitabine was not demonstrated to be therapeutically useful compared with standard treatment [27]. However, that study also showed an interaction between hormone-receptor status and treatment response, suggesting a relation between the response to oral 5-FU and hormone receptors. Several reasons may explain why these drugs were not shown to be useful for postoperative chemotherapy in breast cancer. First, the treatment period was short (duration of treatment with UFT in clinical trials, about 2 years), and the study protocols differed with respect to the timing of endocrine therapy (tamoxifen concurrently used in all clinical trials of UFT). Second, doxifluridine and capecitabine are activated by thymidylate phosphorylase in tumors and therefore might not be adequately effective in a postoperative environment associated with only micrometastases and virtually no tumor.

As mentioned above, clinical studies of UFT, an oral 5-FU derivative, have been performed in Japan and suggested that concurrent use of tamoxifen or an aromatase inhibitor is therapeutically useful. Such therapy may become a treatment option for patients who have ER-positive, HER2-negative luminal breast cancer with intermediate or high baseline risks. In particular, UFT is associated with few adverse events and a good QOL, making it an important option for optimal treatment prescribed according to patients’ preferences.

When antitumor activity against MCF-7/Arom 14 cells was compared among S-1, anastrozole, and S-1 plus anastrozole, S-1 plus anastrozole was confirmed to have significantly higher antitumor activity than either S-1 or anastrozole alone [28].

S-1 combined with standard postoperative endocrine therapy may further enhance inhibition of recurrence in patients with ER-positive, HER2-negative primary breast cancer. The POTENT study is ongoing. This study is being performed in patients with an intermediate risk of recurrence for whom standard chemotherapy was not clearly indicated, as well as patients with a high risk of recurrence in whom standard chemotherapy was indicated. In this study, whether to administer standard adjuvant chemotherapy as prior treatment is left to the physicians’ discretion. When the results of this study become available, it will be possible to study the relation between standard chemotherapy given as prior treatment, and the effectiveness of S-1 for preventing recurrence. Regardless of whether the addition of current standard chemotherapy to endocrine therapy is supported or not supported by the results of other ongoing clinical trials, the results of these studies are expected to be highly applicable to clinical practice.

S-1 is an oral drug with a relatively low incidence of adverse events, allowing treatment without compromising patients’ QOL. As compared with new drugs such as molecular targeted agents, S-1 is less expensive and may thus be a cost-effective treatment for breast cancer.

In summary, UFT and other oral preparations of 5-FU were previously used without adequate evidence of effectiveness, but their use is now internationally supported by the results of a series of controlled clinical trials supporting the benefits and safety of these agents for the management of breast cancer. The results of the POTENT trial are awaited.
